# Membrane potentials regulating GPCRs: insights from experiments and molecular dynamics simulations

**DOI:** 10.1016/j.coph.2016.06.011

**Published:** 2016-10

**Authors:** Owen N Vickery, Jan-Philipp Machtens, Ulrich Zachariae

**Affiliations:** 1 Physics, School of Science and Engineering, University of Dundee, Nethergate Dundee DD1 4NH, UK; 2 Computational Biology, School of Life Sciences, University of Dundee, Dow Street, Dundee DD1 5EH, UK; 3 Institute of Complex Systems, Zelluläre Biophysik (ICS-4), Forschungszentrum Jülich, 52428 Jülich, Germany

## Abstract

G-protein coupled receptors (GPCRs) form the largest class of membrane proteins in humans and the targets of most present drugs. Membrane potential is one of the defining characteristics of living cells. Recent work has shown that the membrane voltage, and changes thereof, modulates signal transduction and ligand binding in GPCRs. As it may allow differential signalling patterns depending on tissue, cell type, and the excitation status of excitable cells, GPCR voltage sensitivity could have important implications for their pharmacology. This review summarises recent experimental insights on GPCR voltage regulation and the role of molecular dynamics simulations in identifying the structural basis of GPCR voltage-sensing. We discuss the potential significance for drug design on GPCR targets from excitable and non-excitable cells.

## Highlights

• A wide range of studies have demonstrated that GPCRs sense membrane voltage.
• MD simulations help illuminate the structure and dynamics of the GPCR voltage-sensor.
• We discuss the implications of GPCR voltage-regulation for drug design.

## Graphical abstract

**Figure:**
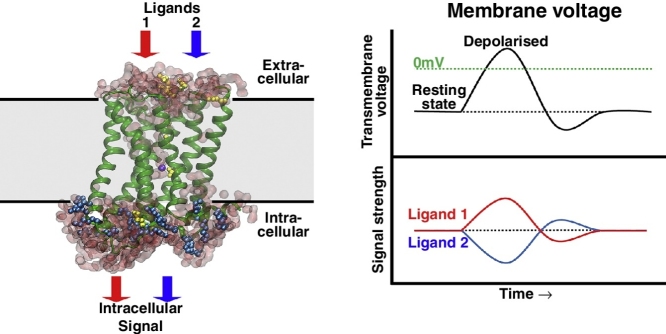

**Current Opinion in Pharmacology** 2016, **30**:44–50

This review comes from a themed issue on **New technologies**

Edited by **Christofer S Tautermann** and **David E Gloriam**

For a complete overview see the Issue and the Editorial

Available online 27th July 2016

**http://dx.doi.org/10.1016/j.coph.2016.06.011**

1471-4892/© 2016 The Authors. Published by Elsevier Ltd. This is an open access article under the CC BY license (http://creativecommons.org/licenses/by/4.0/).

## Introduction

**Figure 1:**
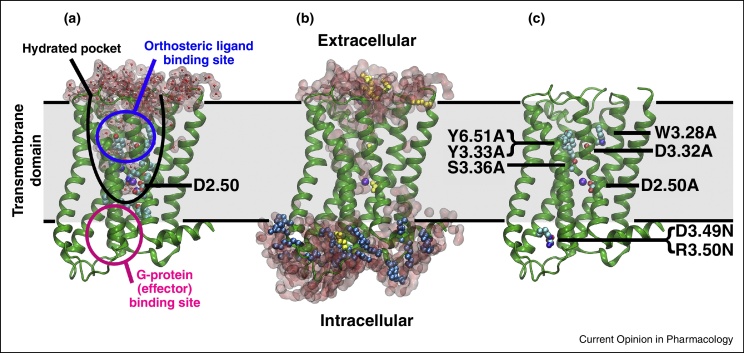
The structural features of class A GPCRs as exemplified by the M_2_ muscarinic receptor. **(a)** The major structural characteristics of class A GPCRs comprise seven transmembrane helices (green), an extracellular ligand binding site (blue circle), an internal hydrated pocket (black), and the intracellular effector protein interaction site (magenta). As both the polar hydrated pocket (water shown in red) and a Na^+^ (purple) ion binding to the charged residue D^2.50^ are conserved amongst class A GPCRs [9^••^], these features are highlighted. The locations of Na^+^ and water in the M_2_ receptor were inferred from MD simulations [10^••^], however the Na^+^ binding site is identical to that observed in crystal structures of other receptors [7, 8••]. **(b)** Distribution of charged residues within the M_2_ receptor (blue: positive; yellow: negative). Most residues, with the exception of three aspartates (D^2.50^, D^3.32^, and D^3.26^) are located outside of the direct influence of the membrane voltage. **(c)** All M_2_ receptor residues that were mutated in Ref [11] to probe the origin of voltage-sensing are shown in cyan. Mutation of these residues was demonstrated to have little or no effect upon gating charges with the exception of D^2.50^A [11].

Membrane proteins form important interfaces mediating the exchange of matter and information between the cell and the external world. They are encoded by about 26% of the human genome [1] and represent a majority both of present as well as potential future drug targets [2]. G-protein coupled receptors (GPCRs) constitute the largest superfamily of membrane proteins in humans with more than 800 members [3]. They transmit binding information of a broad spectrum of extracellular ligands into a range of signalling pathways in the cell [4]. As a consequence, they play a paramount role in therapeutic intervention and are targeted by ∼30% of all presently marketed drugs [5]. Structurally, GPCRs form a bundle of seven transmembrane (TM) helices, which shape a ligand binding site on the extracellular face and an effector binding site on the intracellular side. Within the transmembrane domain, a conserved pocket, which is lined by polar residues and filled with water molecules [6] and a Na^+^ ion [7, 8••, 9••] extends from the ligand binding site towards the effector binding site and almost completely bridges these regions (Figure 1a).

Although the complete mechanism of signal transduction linking ligand binding to activation of the intracellular effector proteins is not yet fully understood, essential elements of this mechanism have been established. There is, for example, ample evidence for conformational changes in the TM domain of the receptors induced by extracellular ligand binding [12]. The changes propagate towards the intracellular side and facilitate the binding of effector proteins, which include a variety of G-proteins and β-arrestins [13]. In the G-protein-dependent signal transduction pathways, ligand binding on the extracellular side leads to the exchange of the nucleotide GDP by GTP in the bound effector G-protein complex. Nucleotide exchange triggers complex dissociation, and the activated G-protein components then transmit the signal to targets residing on the intracellular side [13].

All plasma membranes exhibit a transmembrane potential difference or voltage (*V*_*m*_), generated by electrochemical ion gradients across the bilayer [14]. Like all membrane proteins, GPCRs are therefore located in an environment in which strong electric fields of up to 10^7^–10^8^ V/m exist, as the physiologically relevant voltage gradients drop across the thin hydrophobic core of the membrane, which does not exceed dimensions of ∼3 nm along the membrane normal [15]. Electrically non-excitable cells maintain a resting voltage, which is negative on the intracellular side and undergoes slow oscillations during the cell cycle [14]. In electrically excitable cells — for example, neurons and muscle cells — the coordinated function of voltage-gated ion channels generates action potentials, in which the negative resting voltage displays rapid excursions towards positive values (termed depolarisation). Thus *V*_*m*_ typically adopts values between −90 and +50 mV; however, *V*_*m*_ can reach physiological levels of up to 150 mV, as demonstrated by hair cells in the inner ear [16].

The rapid *V*_*m*_ oscillations typical for action potentials are known to influence the conformation and function of some membrane proteins, an effect that is best understood for voltage-gated ion channels. These channel proteins contain specialised voltage-sensing domains, which are capable of inducing large-scale conformational transitions that gate the channels open or closed, even under small changes of *V*_*m*_ [17]. By contrast, voltage-related effects on other membrane proteins such as GPCRs seem less intuitive, although a number of studies have reported compelling evidence for a broad range of *V*_*m*_-induced phenomena in GPCRs [11, 18••, 19, 20, 21, 22•] (for review see Ref [23]). Many important class A GPCR drug targets are expressed in excitable tissue, for instance the aminergic, opioid, adrenergic and purinergic receptors. Other important excitable tissue GPCRs include the class C metabotropic glutamate receptors, for instance in brain, for which voltage-induced effects have also been reported [24]. Class C GPCRs also have an extended allosteric pocket inside their transmembrane domain, as shown by recent crystal structures [25]. Currently, GPCR voltage regulation has been best characterised for class A GPCRs however, and therefore the focus of this review will be placed on this group. Because of their expression in excitable cells, effects related to *V*_*m*_, and thus the excitation state of the cell, could have an important impact on the function of GPCRs and affect drug action on the receptors. Similarly, slower changes of *V*_*m*_ which have been reported to occur during the cell cycle could play a role in receptor-based signal transduction [14]. The aim of this review is therefore to summarise recent insights on the regulation of GPCRs by *V*_*m*_, discuss its relevance for drug discovery, and highlight the important role of molecular dynamics (MD) simulations in deciphering the dynamic mechanisms of GPCR voltage sensing and their link to GPCR function.

## Experimental evidence for voltage-induced effects in GPCRs

In recent years, *V*_*m*_ has been experimentally demonstrated to affect the conformation, function and transmitted signals of a range of GPCRs [26, 23, 19, 27, 18••]. Voltage-related effects have, for instance, been reported for the muscarinic, adrenergic, and purinergic receptor families [20, 18••, 19]. In most of the earlier work, evidence for voltage regulation was obtained indirectly, and measurements often relied on ionic current through downstream G-protein coupled inward rectifying potassium channels (GIRK) [28] or the use of intracellular calcium-sensitive dyes [26]. Voltage-induced conformational changes in GPCRs have recently also been confirmed directly by FRET-based reporters [18^••^]. Through both GIRK and FRET measurements, it has been shown that voltage can have opposite effects on the transmitted signal induced by agonist action on the receptors [11, 18••, 22•]. For example, the GIRK current elicited by acetylcholine binding to M_2_ receptors in rabbit or feline atrial myocytes is reduced by depolarisation, while that caused by the agonist pilocarpine is strongly enhanced [11, 22•].

The most quantitative measure of voltage-induced rearrangements in GPCRs are electrophysiological recordings, through which gating currents have been determined for several receptor types (Table 1). These transient currents reveal movements of charged regions in membrane proteins, which occur in response to voltage changes. Their name stems from their first observation, caused by the motion of Na^+^ channel voltage sensing domains during the process of channel gating [29]. The electric charge that resides on these voltage sensing domains, usually carried by charged amino acid side-chains, is multiplied by the fraction of the electric field they traverse upon channel gating to give the so-called gating charge. The gating charge can be derived from the gating currents and is expressed in terms of the elementary charge unit [30]. For instance, a singly charged particle moving across 50% of the voltage drop across the membrane would give rise to a gating charge of 0.5*e*.

Gating currents in GPCRs were first recorded for the wild-type (wt) M_2_ muscarinic receptor (M_2_ receptor) and the M_2_ receptor single-mutant (D120^3.49^N)^a^ by cut-open oocyte electrophysiology. In these experiments, gating charges between 0.66 and 0.85*e* were inferred from the observed voltage dependence of the measured gating current (Table 1) [32]. In a more recent study, a gating charge of 0.55*e* on the wt M_2_ receptor was obtained by using the same technique (Table 1 and Figure 1) [11]. Interestingly, a wide range of mutants in which residues of particular interest were modified, including putative ligand binding contacts and conserved charged groups, did neither abolish the recorded gating currents nor markedly alter the observed gating charges [11]. The most prominent exception was the fully conserved residue D69^2.50^ (Figure 1c), which has been identified as the main Na^+^ interacting residue in class A GPCRs [9^••^]. However, it was not clear if this finding, which was obtained before high-resolution crystal structures revealed ion binding in the TM section of GPCRs, resulted from lower surface expression of the mutant or was caused by the mutation itself [11].

Recently, it has been demonstrated by a combination of voltage-clamp and FRET experiments that both G-protein and β-arrestin signalling is strongly modulated by *V*_*m*_ in the muscarinic receptor family [18^••^]. The authors also studied the interplay between ligand action and voltage-induced effects. For instance, they showed that the effect of depolarisation on the transduced signal caused by the agonist carbachol in M_3_ receptors was inverted by a single mutation (N^6.52^Q) within the orthosteric ligand binding site, thereby demonstrating an interaction between the voltage sensor and the ligand binding site. The authors propose that the inversion in voltage sensitivity is due to a changed binding pose of the ligand [18^••^]. Notably, the magnitude of the voltage effect on the signal can be similar to the size of the ligand-induced signal such as in the case of acetylcholine acting upon the M_1_ receptor, as determined by FRET assays probing the arrestin3 signal under depolarisation [18^••^].

## Role of MD simulations in deciphering the structural basis of GPCR voltage-sensing

Most GPCR structures so far have been resolved by X-ray crystallography (for review, see [33•, 34•]). To date, however, it has not yet been possible to experimentally determine membrane protein structures in the presence of a realistic transmembrane voltage. This also currently precludes the direct structural investigation of conformational changes triggered by altered *V*_*m*_.

Present atomistic simulation techniques are commonly capable of modelling membrane proteins in model lipid bilayers over microsecond time spans, allowing MD studies to address many aspects of GPCR function in mechanistic detail. MD simulations have, for instance, been successfully used to shed light on the conformational transition towards the activated receptor state, the role of so-called micro-switches such as the DRY motif (ionic lock), receptor G-protein coupling specificity, nucleotide exchange in the effector complex, internal hydration of the polar pocket, and the processes of ligand attraction and binding [35, 12, 36, 37••, 13]. Voltages across the membrane can be readily included in the MD simulations, either by applying an external electric field 38, 39 or, similar to cells, by imposing TM electrochemical ion gradients 40, 41, as for instance implemented in the Computational Electrophysiology (CompEL) protocol [42^•^].

**Figure 2:**
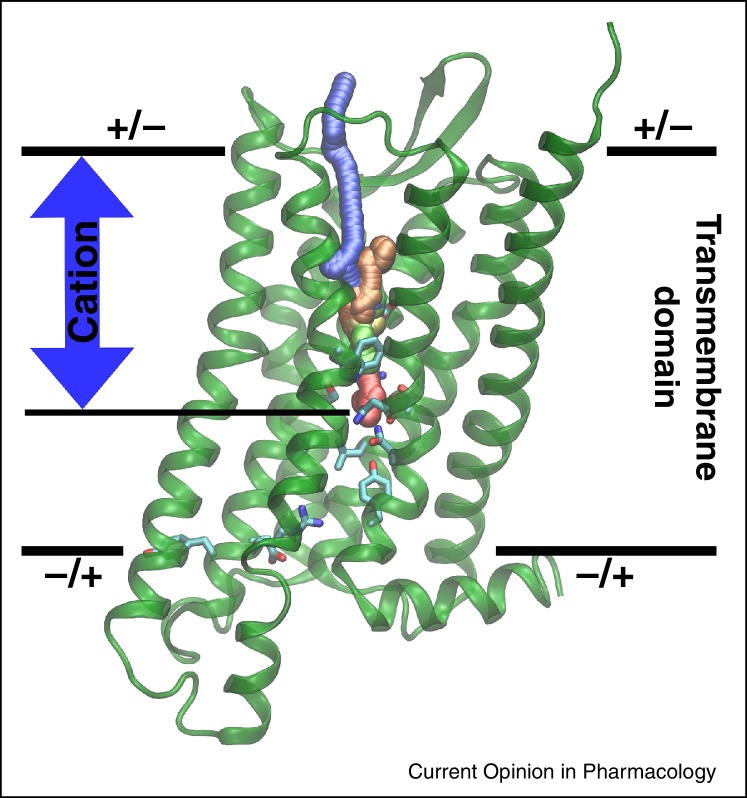
Structural and mechanistic basis of a potential GPCR voltage sensor as derived by MD simulations. Depolarised *V*_*m*_ drives outward migration of an internal cation bound near D^2.50^ towards the extracellular space, crossing the ligand binding pocket. The observed gating charges for this transition are in excellent agreement with experimental values. Upon repolarisation or hyperpolarisation, the cation is attracted back into the allosteric binding pocket. The trajectory of a cation under depolarisation is colour-coded according to the simulation time, proceeding from red to blue.

Recently, voltage-induced conformational changes and the observation of GPCR gating currents have been addressed by using MD simulations. First, a range of supra-physiological *V*_*m*_ were probed by CompEL simulations, followed by a further characterisation of the effects of physiological *V*_*m*_ by free energy calculations. The simulations showed that, in accordance with mutation experiments (Figure 1b,c) [11], none of the charged groups within or near the transmembrane region display substantial voltage-induced motions on the simulation timescales [10^••^]. By contrast, extensive voltage-induced movements of the Na^+^ ion, which binds internally in class A GPCRs to the highly conserved residue D^2.50^ [9^••^], along the water-filled pocket were observed (Figure 2). The movement of this single charge is triggered by depolarised voltages, facilitated by the hydration level of the pocket, and occurs directly within the transmembrane section of the receptor.

Na^+^ has been detected in a range of high-resolution crystal structures of GPCRs [8••, 43, 44, 45•]. Because of the conservation level of D^2.50^ and the polar pocket in general, it is assumed that Na^+^ binding to D^2.50^ is a general feature of class A GPCRs [9^••^]. In addition, Na^+^ is known to have an allosteric effect on the function of most GPCRs [9^••^].

The expected gating charge for the observed movement of a cation from D^2.50^ towards the extracellular entrance of the receptor ligand binding pocket was determined from these MD simulations, and lies in the region of ∼0.53–0.63*e* for Na^+^ in M_2_ receptor and δ-OR variants [10^••^] (see Table 1). Both the observation that ion movement is triggered by depolarisation of *V*_*m*_ and the magnitudes of the gating charges are thus in excellent agreement with the experiments. Moreover, previous MD studies on the δ-OR without applied voltage have demonstrated the internal Na^+^ ion to be mobile, and able to leave the receptor under the influence of an applied force [46]. Importantly, it has also been shown that small organic cations such as amiloride can replace Na^+^ in the pocket under low Na^+^ concentration, exerting an allosteric effect similar to Na^+^ [47^••^]. It is therefore possible that other cations can undergo analogous movements within the pocket upon depolarisation, depending on experimental conditions, and give rise to comparable gating charges [32]. This includes potential protonation changes of the side chain of D^2.50^, during which a proton could be exchanged with the external solution [10^••^] (Table 1).

## Implications for GPCR physiology and pharmacology

Na^+^ plays a central role in GPCR function, shifting the equilibrium between active and inactive receptors, regulating agonist binding, and biasing downstream signals [9••, 8••]. Any voltage-dependence of the occupancy of the GPCR allosteric pocket with Na^+^ or its position within the receptors could therefore have major functional implications for signal transduction and signal bias. Alongside its potential structural basis, voltage-dependence of GPCR conformation or signalling has now been established for a range of GPCRs and deserves the attention of drug designers and pharmacologists alike. As shown by Rinne *et al.* [18^••^], voltage can either enhance or attenuate the transmitted agonist signal depending on the ligand and precise environment of the binding site. In addition, ligand binding affinity has been shown to be voltage-dependent [32].

Electrophysiological properties such as resting *V*_*m*_ vary substantially between different cell types. For example, neurons display a markedly shifted *V*_*m*_ in various brain regions and developmental stages [48]. The action of GPCR ligands is therefore likely to depend on the cellular context. In electrically excitable cells, the transduced GPCR signal could also be altered by the excitation state of the cell. It has for instance been demonstrated that GPCR voltage sensing modulates synaptic neurotransmission by reshaping the kinetics of voltage-dependent transmitter release on the millisecond timescale [49]. Therefore, it is conceivable that GPCRs can establish dynamic feedback routes, by which voltage information is transmitted back into a range of intracellular signals on both fast and slow timescales. Notably, recent cancer research has revealed that a range of malignant cell types possess a more depolarised resting voltage than quiescent cells [50•, 14, 51]. Although GPCRs have traditionally received less attention than other proteins as cancer drug targets, GPCRs are known to be involved in cancer initiation and progression [52^•^]. The role of GPCR voltage regulation has, to our best knowledge, however not yet been investigated in this context.

Similarly, it has recently been demonstrated that oncogenic signalling pathways are influenced by *V*_*m*_ through the redistribution of charged lipids in the inner leaflet of the plasma membrane [50^•^]. Because membrane lipids allosterically modulate GPCR activity [53^•^], *V*_*m*_ could thus also have an indirect impact on receptor signal transduction via an effect on lipid distribution. As we only begin to appreciate the importance of *V*_*m*_ in regulating membrane proteins either directly or indirectly, much further work is needed to fully understand the role of *V*_*m*_ in GPCR signalling and its implications for the drug design process, which could be wide-ranging.

## Conclusions

Recent experimental and computational insights suggest that the membrane voltage has an important impact on GPCR pharmacology. In particular, MD simulations under voltage are able to characterise functionally important movements in GPCRs driven by potential differences. Further simulations would be useful to investigate the interplay of voltage-induced changes with ligand binding and signal transduction. The fact that GPCRs are voltage-sensitive, together with its possible structural underpinning, should be taken into consideration during drug development on GPCR targets, as especially in excitable cell GPCRs, voltage-sensing could be an important mechanism of feeding back voltage information into intracellular signal transduction pathways. Thereby, the signal that is actually induced by a ligand might depend on the excitation state of the cell, which would have important consequences for drug discovery on excitable tissue GPCRs. It should also be investigated if agonists can show variations in their effect on different cell types, including non-excitable cells, owing to a difference in resting *V*_*m*_.

## Conflict of interest statement

Nothing declared.

## References and recommended reading

Papers of particular interest, published within the period of review, have been highlighted as:

• of special interest

•• of outstanding interest

## Figures and Tables

**Table 1:** Measured and calculated gating charges of class A GPCRs.

Receptor	Gating charge (*e*)	Reporter method	Refs
m1 muscarinic:			
wt	0.72, 0.76^a^	FRET	[18^••^]
m2 muscarinic:			
wt	0.55	Electrophysiology	[11]
wt	0.53 (Na^+^)	MD simulation	[10^••^]
wt	0.52 (proton)	MD simulation	[10^••^]
wt	0.7, 0.85	Electrophysiology	[32]
D69^2.50^A	NR	Electrophysiology	[11]
W99^3.28^A	0.8	Electrophysiology	[11]
D103^3.32^A	0.5	Electrophysiology	[11]
Y104^3.33^A	0.54	Electrophysiology	[11]
S107^3.36^A	0.49	Electrophysiology	[11]
D120^3.49^N	0.66	Electrophysiology	[32]
D120^3.49^N-R120^3.50^N	0.52	Electrophysiology	[11]
D120^3.49^N-R120^3.50^N	NR	Electrophysiology	[32]
Y403^6.51^A	0.57	Electrophysiology	[11]
α_2*A*_-adrenergic			
wt	0.5	FRET	[19]
δ-opioid:			
wt	0.42 (Na^+^)	MD simulation	[10^••^]
N131^3.35^V	0.63 (Na^+^)	MD simulation	[10^••^]

NR, not resolved.

a Precise value depends on methodology used.

## Footnotes

a Superscripts refer to the Ballesteros–Weinstein generic residue numbering nomenclature [31].
